# Isolated Fracture of the Coracoid Process

**DOI:** 10.1155/2014/482130

**Published:** 2014-01-06

**Authors:** Ali Güleç, Harun Kütahya, Recep Gani Göncü, Serdar Toker

**Affiliations:** ^1^Department of Orthopedics and Traumatology, Konya Training and Research Hospital, Konya, Turkey; ^2^Department of Orthopedics and Traumatology, Konya Beyhekim State Hospital, Selçuklu, 42100 Konya, Turkey; ^3^Department of Orthopedics and Traumatology, Mevlana University Medical Faculty, Konya, Turkey; ^4^Department of Orthopedics and Traumatology, Meram Medical School, Necmettin Erbakan University, Konya, Turkey

## Abstract

Coracoid fractures are rarely seen fractures. In the shoulder girdle, coracoid process fractures generally accompany dislocation of the acromioclavicular joint or glenohumeral joint, scapula corpus, clavicula, humerus fracture, or rotator cuff tear. Coracoid fractures can be missed and the treatment for coracoid process fractures is still controversial. In this paper, a 34-year-old male manual labourer presented to the emergency department with complaints of pain and restricted movement in the left shoulder following a traffic accident. On direct radiographs and computerised tomography images a fragmented fracture was observed on the base of the coracoid process. In addition to the coracoid fracture, a mandibular fracture was determined. The patient was admitted for surgery on both fractures. After open reduction, fixation was made with a 3.5 mm cannulated screw and washer. At the postoperative 6th week, bone union was determined. The patient returned to his previous occupation pain-free and with a full range of joint movement. In conclusion, in the current case of isolated fragmented coracoid process fracture showing minimal displacement in a patient engaged in heavy manual work, surgery was preferred as it was thought that nonunion might be encountered particularly because of the effect of forces around the coracoid.

## 1. Introduction

Coracoid fractures are rarely seen fractures [1]. In the shoulder girdle, coracoid process fractures generally accompany dislocation of the acromioclavicular (AC) joint or glenohumeral joint, scapula corpus fracture, clavicular fracture, humerus proximal end fracture, or rotator cuff tear [2]. Coracoid fractures can be missed and the treatment for coracoid process fractures is still controversial. The case presented here is of an isolated coracoid process fracture treated surgically.

## 2. Case Report

**Figure 1:**
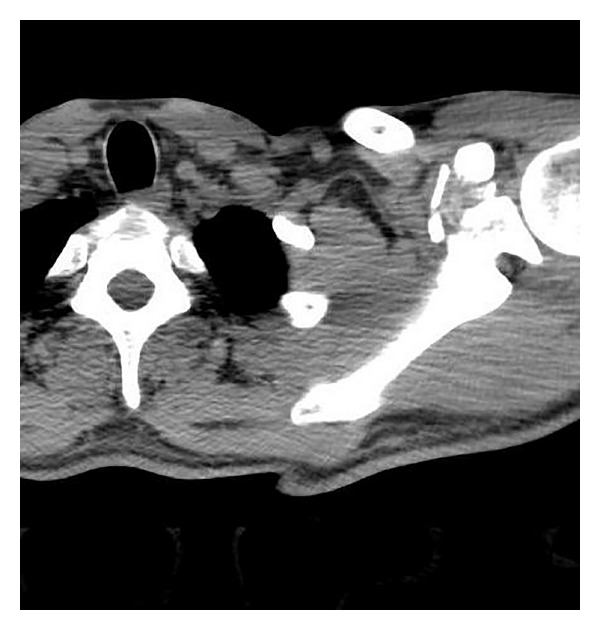
Preoperative computerised tomography (CT) image.

**Figure 2:**
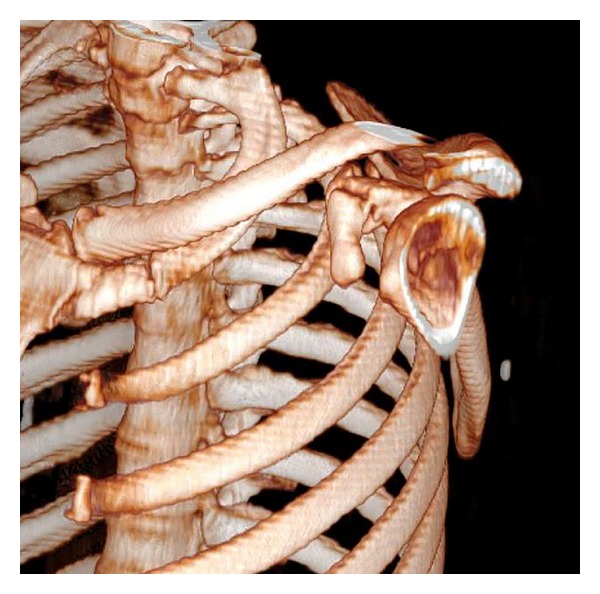
Preoperative 3-dimensional CT image.

**Figure 3:**
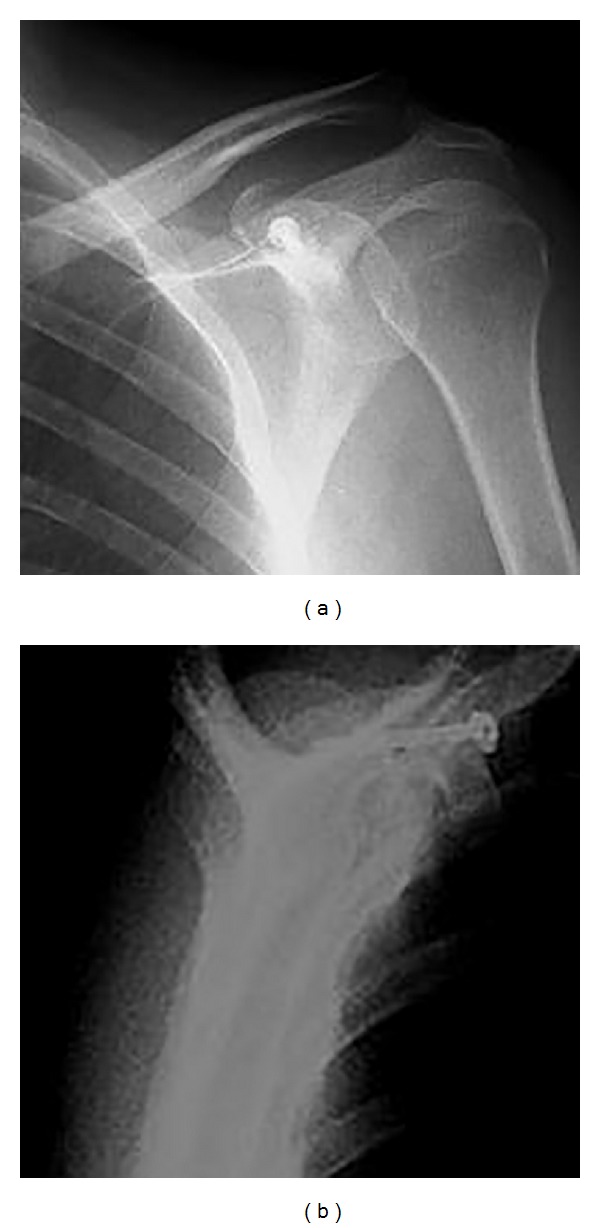
Postoperative 6th week X-ray images (fracture fixed bicortical).

A 34-year-old male manual labourer presented at the Emergency Department with complaints of pain and restricted movement in the left shoulder following a traffic accident. In the physical examination, ecchymosis and sensitivity in the left shoulder, restricted shoulder movements, and sensitivity in the jaw were determined. The results of the neurovascular examination were normal. On direct radiographs and computerised tomography (CT) images a fragmented fracture was observed on the base of the coracoid process (Figures 1 and 2). In addition to the coracoid fracture, a mandibular fracture was determined. The patient was admitted for surgery on both fractures. After making the incision along the Langer's line on the coracoid process, the fracture line was reached. After open reduction, fixation was made with a 3.5 mm cannulated screw and washer. Postoperatively, the patient was followed up for 2 weeks with the application of a simple shoulder sling. Passive joint exercises were allowed in the first 2 weeks; from the 3rd postoperative week, active joint movement exercises were started and from the 5th week, shoulder strengthening exercises. At the postoperative 6th week, bone union was determined (Figure 3). The patient returned to his previous occupation pain free and with a full range of joint movement.

## 3. Discussion

Isolated coracoid fractures are seen extremely rarely. All coracoid process fractures constitute approximately 1% of all fractures and 2–13% of scapula fractures [3–5]. Fractures are often seen on the base of the coracoid process and are generally minimally displaced and together with AC joint injuries [6].

Coracoid fractures may be easily missed. Lal et al., in a case report where surgery had not been applied and Vaienti and Pogliacomi in a series of 9 cases with delayed diagnosis, applied conservative treatment and clearly demonstrated this situation [7, 8]. In cases which cannot be determined with direct radiographs, the use of CT may be necessary. In a study by Botchu et al. of 7 cases, it was shown that coracoid process fractures can be diagnosed with ultrasonography [9]. In the case presented here, diagnosis was made with CT.

An important point related to coracoid fractures is the neurological injuries which may accompany the fracture. Neer stated that in fractures involving the coracoid process in particular, there may be brachial plexus pressure and suprascapular nerve paralysis and therefore evaluation with electromyography prior to exploration is recommended [10].

Treatment of coracoid process fractures has not yet achieved clarity. The majority is preferably treated conservatively [6]. Previous studies on this subject have mostly been in the form of a case report or series of no relevance to others. The most extensive study in literature related to coracoid process fractures was conducted by Anavian et al. Surgery was applied to 14 coracoid process fractures of 26 patients including scapula process fractures and successful results were obtained for all patients [11].

Indications for surgical treatment were accepted as painful nonunion, >1 cm displacement, concomitant scapula fracture on the same side and the presence of superior shoulder suspensory complex injuries. In a study by Lal and Bansal of 22 patients, all with coracoid fractures and various shoulder girdle injuries, 10 patients were treated conservatively and nonunion was encountered in 1 patient [8]. Spormann et al. operated on 3 cases of isolated coracoid process fracture and obtained successful results [12]. Again successful results were obtained from surgical treatment applied by Subramanian et al. of an isolated coracoid fracture in an unstable shoulder [13]. Garcia-Elias and Salo applied excision following shoulder dislocation and reported nonunion of the coracoid process [14]. In studies by Guttentag and Rechtine and Goos, conservative treatment was applied to coracoid fractures in athletes and patients engaged in heavy manual work and poor results were obtained [15, 16]. In the current case, as the patient was a construction worker, surgery was preferred despite the minimal displacement and successful results were obtained.

In coracoid fractures, surgical fixation can be applied with open reduction and with screws [12]. Even though the most frequently used method is the anterior approach, indirect reduction and fixation may be applied with a posterior approach [11]. In a study by Bhatia, fluoroscopy-guided percutaneous fixation was applied to a coracoid process fracture which was accompanied by AC joint dislocation [17]. In the current case, fixation was achieved with 1 screw and washer following open reduction with an anterior approach.

In conclusion, in the current case of isolated fragmented coracoid process fracture showing minimal displacement in a patient engaged in heavy manual work, surgery was preferred as it was thought that nonunion might be encountered particularly because of the effect of forces around the coracoid. Although this is a rarely seen fracture, further multicentre, randomised controlled studies would give clearer ideas about the choice of treatment alternatives.
